# The top tertile of hematocrit change during hospitalization is associated with lower risk of mortality in acute heart failure patients

**DOI:** 10.1186/s12872-017-0669-0

**Published:** 2017-09-02

**Authors:** Haobin Zhou, Tianyu Xu, Yuli Huang, Qiong Zhan, Xingfu Huang, Qingchun Zeng, Dingli Xu

**Affiliations:** 1 State Key Laboratory of Organ Failure Research, Department of Cardiology, Nanfang Hospital Southern Medical University, 1838 North Guangzhou Avenue, Guangzhou, 510515 China; 2 State Key Laboratory of Cardiovascular Disease, Heart Failure Center, Fuwai Hospital, National Center for Cardiovascular Diseases, Chinese Academy of Medical Sciences and Peking Union Medical College, 167 Beilishi Road, Beijing, 100037 China; 3 First School of Clinical Medicine, Southern Medical University, Guangzhou, 510515 China

**Keywords:** Hematocrit, Hemoconcentration, Follow-up, All-cause death, Acute heart failure

## Abstract

**Background:**

Hemoconcentration has been proposed as surrogate for changes in volume status among patients hospitalized with acute heart failure (AHF) and is associated with a favorable outcome. However, there is a dearth of research assessing the clinical outcomes of hospitalized patients with hemoconcentration, hemodilution and unchanged volume status.

**Methods:**

We enrolled 510 consecutive patients hospitalized for AHF from April 2011 to July 2015. Hematocrit (HCT) levels were measured at admission and either at discharge or on approximately the seventh day of admission. Patients were stratified by delta HCT tertitles into hemodilution (ΔHCT ≤ − 1.6%), no change (NC, −1.6% < ΔHCT ≤1.5%) and hemoconcentration (ΔHCT >1.5%) groups. The endpoint was all-cause death, with a median follow-up duration of 18.9 months.

**Results:**

Hemoconcentration was associated with lower left ventricle ejection fraction, as compared with NC and hemodilution groups, while renal function at entry, New York Heart Association class IV, and in-hospital worsening renal function (WRF) were not significantly different across the three groups. After multivariable adjustment, hemoconcentration had a lower risk of mortality as compared with hemodilution [hazard ratio (HR) 0.39, 95% confidence interval (CI) 0.24–0.63, *P* < 0.001], or NC (HR 0.54, 95% CI 0.33–0.88, *P* = 0.015], while hemodilution and NC did not have significantly differ in mortality (HR 0.72, 95% CI 0.48–1.10, *P* = 0.130).

**Conclusions:**

In patients hospitalized with AHF, an increased HCT during hospitalization is associated with a lower risk of all-cause mortality than a decreased or unchanged HCT. Furthermore, all-cause mortality does not differ significantly between patients with unchanged and decreased HCT values.

## Background

Acute heart failure (AHF) is a leading cause of hospitalization among patients ≥65 years of age worldwide and has deleterious impacts on healthcare costs and quality of life [1–4].

Data from both the ADHERE (Acute Decompensated Heart Failure Registry), and OPTIMIZE-HF (Organized Program to Initiate Lifesaving Treatment in Hospitalized Patients with Heart Failure) registries have demonstrated that in the majority of patients admitted with AHF the primary cause is volume overload [5, 6]. Accordingly, optimal volume management in AHF patients is crucial yet challenging due to the complex underlying pathophysiology of the disease. In the setting of decompensated heart failure, the primary objective is to remove the excessive intravascular and extravascular fluid, and relieve congestive signs and symptoms [7]. Hemoconcentration, defined as an increase in hemoglobin (Hgb), hematocrit (HCT) or plasma albumin, has been suggested as an appropriate surrogate for the assessment of changes in volume status [8, 9].

Previous data has demonstrated the prognostic impacts of hemoconcentration in patients hospitalized with AHF [8–13]. Testani et al. [8] and Van der Meer et al. [9] reported that among patients with AHF, those with hemoconcentration experienced better survival compared to those without, although at the expense of an increased worsening renal function (WRF) rate. Subsequently, Testani et al. demonstrated that the subset of patients experiencing late hemoconcentration during hospitalization achieved better survival [14]. More recently, ter Maaten et al. found that combining two markers of decongestion, namely hemoconcentration and diuretic response, improves risk prediction for early rehospitalization for AHF and may provide an easily accessible tool for clinicians to identify low-risk patients [15].

However, the definition of hemoconcentration has become controversial and variable criteria have been used in earlier studies. In the ESCAPE (The Evaluation Study of Congestive Heart Failure and Pulmonary Artery Catheterization Effectiveness) Trial, hemoconcentration was diagnosed when ≥2 test values among delta HCT, delta total protein and delta albumin (between baseline and discharge) were in the highest tertile [8], while in the EVEREST (The Efficacy of Vasopressin Antagonism in Heart Failure Outcome Study with Tolvaptan) trial, hemoconcentration was defined by the top quartile of HCT change between baseline and discharge or day 7 (whichever occurred first) [12]. Van der Meer et al. and ter Maaten et al. defined hemoconcentration using Hgb, specifically, an increased Hgb level from admission to day 7 or discharge [9, 15]. Additionally, it was defined as an absolute increase in both HCT and Hgb levels above admission values at any time during hospitalization in an early study by Testani et al. [14]. These studies have most often assessed changes in volume status in the setting of hemoconcentration and the absence of hemoconcentration. However, whether patients with a largely unchanged or decreased HCT values (no change in volume status or hemodilution) experience the same mortality risks or benefits as those with hemoconcentration respectively remained unclear. Little data is available comparing the clinical outcomes of hospitalized patients with hemoconcentration, hemodilution and unchanged volume status.

As such, the objective of this study was to assess the effect of tertiles of HCT change over time on all-cause mortality in patients admitted with AHF.

## Methods

### Study design

We performed a retrospective chart review of consecutive patients admitted to Nanfang hospital, Southern Medical University between April 2011 and July 2015 with a primary discharge diagnosis of AHF and documented ejection fraction on admission. Baseline data were obtained through an electronic medical recording system, wherein demography, physical examination, laboratory tests, echocardiography, medical history, prescriptions and medication related data were recorded. AHF was defined according to the Chinese Society of Cardiology guidelines on heart failure (HF) [16, 17] as a rapid onset of symptoms and signs secondary to abnormal cardiac function requiring urgent treatment. Additional inclusion criteria included a baseline N-terminal pro-brain natriuretic peptide (NT-proBNP) level > 300 pg/mL, age ≥ 18 years and a length of stay ≥3 days (patients with time spent in hospital <3 days may experience inadequate decongestion). Patients with acute myocardial infarction, malignant hematological diseases, severe anemia (Hgb <70 g/L), end stage kidney disease (requiring renal replacement therapy), malignancy and active bleeding were excluded. Patients who underwent blood transfusion, erythropoietin therapy or iron replacement therapy during hospitalization and those without follow-up data were also excluded. For those with multiple hospitalizations, only data from the first hospitalization were included for analysis. This study was approved by the institutional review board of Nanfang Hospital.

Venous blood samples for measurements of HCT were taken within 24 h of admission (baseline) and either at discharge or on approximately the seventh day postadmission (whichever occurred first, second timepoint). For the second timepoint, HCT values obtained outside the 3 to 10 day postadmission window were excluded. Furthermore, patients with no HCT data at either time point were excluded. The relative change between baseline and second timepoint HCT values were calculated (ΔHCT). To assess the association between HCT change and outcome, the patient population was stratified into three groups based on delta HCT tertiles: hemodilution (decreased, ΔHCT ≤ − 1.6%), no change (NC, unchanged, −1.6% < ΔHCT ≤1.5%) and hemoconcentration (increased, ΔHCT >1.5%).

Estimated glomerular filtration rate (eGFR) was calculated using the Modification of Diet in Renal Disease eq. [18]. WRF was defined as an increase in serum creatine of ≥0.3 mg/dl (≥26.4 umol/L) at any time from baseline to the second timepoint [10, 11]. Anemia was defined as Hgb <13 g/dl for men and <12 g/dl for women, according to the World Health Organization (WHO) criteria.

Survival information was obtained directly from patients or their relatives by telephone, or from the electronic medical recording system of the hospital up to April 1, 2016. The primary endpoint was all-cause mortality. Data regarding HF rehospitalizations was not available in the current study. Given the China-specific medical insurance policies, using rehospitalization as a parameter to assess HF outcomes may not be accurate in a retrospective study.

### Statistical analysis

Normally distributed continuous variables were presented as mean ± SD, while non-normal data was presented by the median and interquartile range (IQR). Categorical variables were presented as percentages. Differences in characteristics across delta HCT groups were compared with the Chi-sqaure test for categorical data and the ANOVA or Kuskal-Wallis for continuous data, as appropriate. Kaplan–Meier curves were used to evaluate survival over time and the log-rank test was used to assess differences in survival across delta HCT groups. Cox proportional hazards models were used to evaluate the impact of HCT change on all-cause mortality. Cox models fulfilled the proportional-hazards assumption (using R). All the variables at entry which were statistically significant (*P* < 0.20) at univariate analysis were entered in the model. Variables with >10% missing values (body mass index, WRF) were not included in the univariate analysis. Baseline HCT and anemia were not included in the model owing to their significant correlation with Hgb (*r* = 0.97, *p* < 0.001; *r* = −0.84, *p* < 0.001). Backward selection was used. Hazard ratios (HRs) with 95% confidence intervals (CIs) were calculated. Sample size analysis was performed using PASS version 11 software found a minimum sample size of 486 was sufficient (power = 0.8, alpha = 0.025).

A *P* value <0.05 was considered statistically significant. All tests were two-sided. All analyses were performed using SPSS version 20.0 (IBM SPSS Statistics, IBM Corporation, Armonk, New York).

## Results

**Fig. 1:**
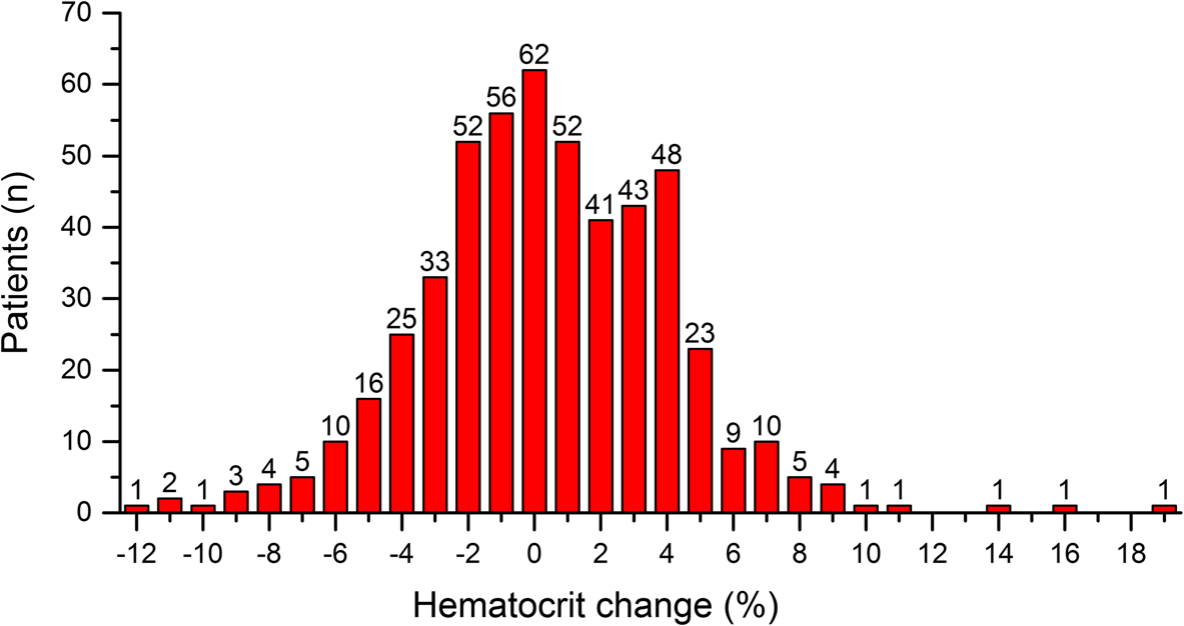
Distribution of hematocrit change in 510 patient hospitalized with AHF

A total of 510 AHF patients (66.2 ± 14.1 years; 38.0% women) meeting inclusion criteria were enrolled in this study. The distribution of delta HCT levels is shown in Fig. 1.

Table 1 shows the baseline characteristics of the overall population stratified by tertiles of HCT change. Hemoconcentration patients had a higher heart rate at entry and a lower LVEF as compared to those with hemodilution and NC. Baseline Hgb, HCT, eGFR, New York Heart Association (NYHA) class IV and WRF in-hospital appeared to be comparable across delta HCT categories. Medical histories in hemoconcentration were largely similar to those in the other two groups. The primary differences were that the hemoconcentration strata had a higher prevalence of atrial fibrillation and a lower prevalence of ischemic heart disease.

**Table 1:** Baseline characteristics of AHF patients according to tertiles of hematocrit change from baseline to discharge or close to day 7

	Total	Hemodilution	No change	Hemoconcentration	
		ΔHCT ≤ − 1.6% -1.6% < ΔHCT ≤1.5% ΔHCT >1.5%			
Variables	(*n* = 510)	(*n* = 171)	(*n* = 170)	(*n* = 169)	*P*-value
Demographics					
Age, years	66.2 ± 14.1	66.6 ± 14.0	66.4 ± 14.5	65.7 ± 13.9	0.817
Female	194 (38.0)	61 (35.7)	59 (34.7)	74 (43.8)	0.167
Smoker	159 (31.2)	59 (34.5)	51 (30.0)	49 (29.0)	0.505
Admission physical examination					
Systolic BP, mm Hg	132.0 ± 26.3	133.6 ± 27.8	131.2 ± 27.1	131.1 ± 23.7	0.602
Diastolic BP, mm Hg	78.5 ± 15.7	77.5 ± 15.4	77.4 ± 16.3	80.6 ± 15.4	0.109
Heart rate, beats/min	88.0 (73.8–100.0)	88.0 (75.0–100.0)	84.0 (72.0–96.0)	90.0 (75.0–111.0)	0.010
BMI, kg/m^2^	23.0 ± 4.0	22.9 ± 4.3	22.7 ± 3.8	23.4 ± 3.8	0.240
NYHA class IV	155 (76.5)	42 (69.6)	53 (72.9)	60 (87.0)	0.087
Echocardiography data					
LVEDD, mm	52.5 ± 10.5	50.3 ± 10.1	52.3 ± 10.5	55.1 ± 10.4	<0.001
LVEF, %	48.6 ± 12.3	49.9 ± 11.7	49.2 ± 12.3	46.6 ± 12.7	0.030
Laboratory values					
Plasma potassium, mmol/L	4.1 ± 0.6	4.1 ± 0.6	4.1 ± 0.6	4.1 ± 0.7	0.479
Serum sodium, mmol/L	140.0 (137.0–142.0)	139.0 (136.0–142.0)	140.0 (136.0–142.0)	140.0 (138.0–142.5)	0.026
Uric acid, mg/dL	5.4 ± 1.8	5.2 ± 1.8	5.5 ± 1.7	5.5 ± 1.9	0.285
eGFR, ml/min/1.73m^2^	59.3 (42.2–76.2)	56.6 (41.3–73.2)	56.5 (40.1–75.0)	62.7 (43.8–79.7)	0.150
HsCRP, mg/L	7.6 (3.0–20.0)	7.6 (2.6–32.0)	6.0 (2.3–15.8)	9.4 (3.8–24.5)	0.017
NT-proBNP, pg/mL	4115.0 (1601.8–8479.0)	3462.0 (1357.0–7161.0)	3638.0 (1484.3–8353.0)	4731.0 (2153.0–10,455.5)	0.052
Hemoglobin, g/dL	12.6 ± 2.3	12.8 ± 2.5	12.6 ± 2.3	12.5 ± 2.0	0.539
Hematocrit, %	38.0 ± 6.3	38.5 ± 6.8	37.8 ± 6.4	37.8 ± 5.8	0.462
Albumin, g/dL	3.6 ± 0.5	3.6 ± 0.6	3.6 ± 0.5	3.5 ± 0.5	0.043
LDL-c, mmol/L	2.6 ± 0.9	2.8 ± 1.0	2.5 ± 0.9	2.4 ± 0.8	0.003
Medical history					
Hypertensive etiology	123 (24.1)	47 (27.5)	37 (21.8)	39 (23.1)	0.433
Ischemic heart disease	201 (39.4)	71 (41.5)	76 (44.7)	54 (32.0)	0.044
Dilated cardiomyopathy	95 (18.6)	53 (31.0)	46 (27.1)	36 (21.3)	0.126
Valvular heart disease	76 (14.9)	18 (10.5)	25 (14.7)	33 (19.5)	0.066
Diabetes mellitus	135 (26.5)	53 (31.0)	46 (27.1)	36 (21.3)	0.126
Stroke	54 (10.6)	18 (10.5)	17 (10.0)	19 (11.2)	0.933
Anemia	245 (48.0)	81 (47.4)	88 (51.8)	76 (45.0)	0.446
WRF	81 (18.2)	31 (21.1)	31 (20.5)	19 (13.0)	0.135
Atrial fibrillation	166 (32.5)	50 (29.2)	47 (27.6)	69 (40.8)	0.018
COPD	38 (7.5)	18 (10.5)	8 (4.7)	12 (7.1)	0.120
Medications at discharge					
Loop diuretic	356 (76.1)	111 (72.1)	108 (70.6)	137 (85.1)	0.004
ACE-I/ARB	307 (65.6)	95 (61.7)	105 (68.6)	107 (66.5)	0.424
Beta-blocker	259 (55.3)	89 (57.8)	83 (54.2)	87 (54.0)	0.756
Aldosterone antagonist	346 (73.9)	103 (66.9)	111 (72.5)	132 (82.0)	0.008
Digoxin	71 (15.2)	16 (10.4)	13 (8.5)	42 (26.1)	<0.001

*BP* blood pressure, *BMI* Body mass index, *NYHA class* New York Heart Association, *eGFR* estimated glomerular filtration rate, *LVEF* left ventricle ejection fraction, *LVEDD* left ventricular end-diastolic diamater, *HsCRP* hgih-sensitivity C-reactive protein, *NT-proBNP* N-terminal pro-brain natriuretic peptide, *LDL-c* low density lipoprotein cholesterol, *WRF* worsening renal function, *COPD* chronic obstructive pulmonary disease, *ACE-I/ARB* angiotensin-converting enzyme inhibitor/angiotensin receptor blocker

Values are mean ± SD, n (%), or median (interquartile range)

**Fig. 2:**
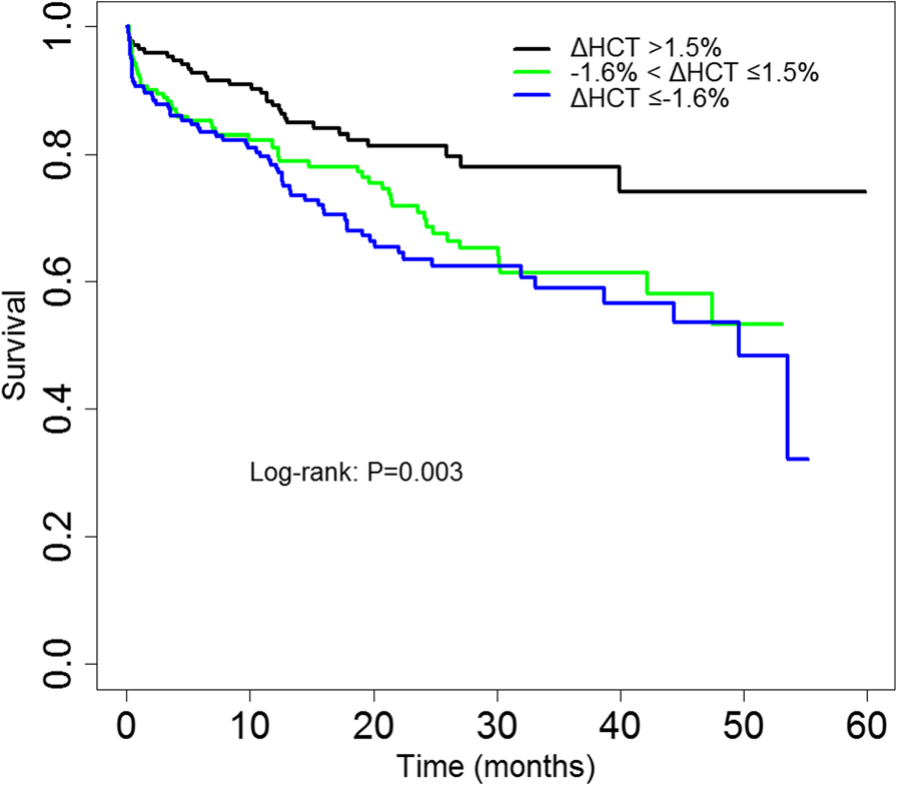
Kaplan-Meier survival curves for all-cause mortality. Curve according to tertiles of delta HCT (tertile 1, ΔHCT ≤ − 1.6%; tertile 2, −1.6% < ΔHCT ≤1.5%; tertile 3, ΔHCT >1.5%)

There were 147 (28.8%) all-cause deaths over a median follow-up period of 18.9 months (IQR 11.5–30.0 months). The number of deaths were 62 (36.3%), 54 (31.8%), and 31 (18.3%) in the hemodilution, NC, and hemoconcentration groups respectively. In Kaplan-Meier survival analyses (Fig. 2), patients with hemoconcentration had the best clinical outcomes, in contrast to those with NC, and hemodilution (log-rank *P* = 0.003). Survival differences in the pairwise comparison were: hemodilution versus NC (*P* = 0.407), hemodilution versus hemoconcentration (*P* = 0.001), NC versus hemoconcentration (*P* = 0.013). In univariate models (Table 2), there was an increased risk of mortality for hemodilution [hazard ratio (HR) 2.09, 95% confidence interval (CI) 1.35–3.21, *P* = 0.001] and NC (HR 1.78, 95% CI 1.14–2.77, *P* = 0.011) compared with hemoconcentration. Mortality did not differ between patients with NC and hemodilution (HR 0.85, 95% CI 0.59–1.23, *P* = 0.390). In multivariate Cox proportional hazard models adjusted for confounders (age, smoking, diastolic blood pressure, NYHA class, plasma potassium, serum sodium, uric acid, eGFR, high-sensitivity C-reactive protein (log), NT-pro-BNP (log), Hgb, albumin, low density lipoprotein cholesterol (LDL-c), dilated cardiomyopathy, valvular heart disease, diabetes mellitus, stroke, chronic obstructive pulmonary disease, loop diuretics, ACE-I/ARBs, beta-blockers) hemoconcentration had a lower risk of mortality as compared with hemodilution [hazard ratio (HR) 0.39, 95% confidence interval (CI) 0.24–0.63, *P* < 0.001], or NC (HR 0.54, 95% CI 0.33–0.88, *P* = 0.015]. However, the mortality in NC did not differ significantly from that in hemodilution (HR 0.72, 95% CI 0.48–1.10, *P* = 0.130).

**Table 2:** Univariate and multivariable predictors of all-cause mortality in patients with acute heart failure

	Hemodilution	No change	Hemoconcentration
Unadjusted HR (95% CI) *P* value	1 (referent)	0.85(0.59–1.23) 0.390	0.48(0.31–0.74) 0.001
Adjusted HR^a^ (95% CI) *P* value	1 (referent)	0.72(0.48–1.10) 0.130	0.39(0.24–0.63) <0.001

*HR* hazard ratio, *CI* confidence interval; other abbreviations as in Table 1

^a^age, smoking, diastolic blood pressure, NYHA class, plasma potassium, serum sodium, uric acid, eGFR, high-sensitivity C-reactive protein (log), NT-pro-BNP (log), Hgb, albumin, low density lipoprotein cholesterol (LDL-c), dilated cardiomyopathy, valvular heart disease, diabetes mellitus, stroke, chronic obstructive pulmonary disease, loop diuretics, ACE-I/ARBs, beta-blockers were included in the multivariate Cox regression models

## Discussion

This study demonstrates that in patients hospitalized with AHF the change in HCT over time is a powerful predictor of mortality independent of other prognostic markers. An increase in HCT concentration during hospitalization is associated with better survival rates as compared with NC or a decreased HCT concentration. However, there is no difference in the risk of mortality among those who experience unchanged or decreased HCT concentrations. These findings suggest that HCT, a simple and widely available parameter, can be useful in the clinical settings to identify patients with possible adverse outcomes and serve as a potential target for therapeutic intervention.

During physiological conditions, the fluid flow inside and outside the vessel maintains a dynamic equilibrium. When intravascular fluid is removed faster than it can be replaced by fluid from the extravascular space, hemoconcentration occurs [7, 8, 14]. Various approaches exist for the evaluation of volume status including evaluation of physical signs and symptoms (jugular venous pressure, dyspnea, S3, etc.), pulmonary artery catheterization parameters (pulmonary capillary wedge pressure, central venous pressure) and tracer techniques (i.e., I^131^-tagged albumin). Nevertheless, these methods are often invasive, expensive, time consuming and lack specificity and sensitivity. Indirect methods (HCT, protein, and albumin level etc.) that are widely available and inexpensive have been proposed to estimate changes in volume status despite their limitations [19, 20]. Thus far, although various surrogates to assess changes in volume status are available, no standardized criteria for volume evaluation have been defined. In the present study, we chose to use HCT change to define volume change rather than other parameters because it is simple and widely available, of significant clinical value and its use is already established by nephrologists for the monitoring of circulating blood volume status during hemodialysis [21, 22].

Previous studies comparing patients hospitalized for HF with hemoconcentration to those without described a link between hemoconcentration and favorable outcomes [8–13]. Interestingly, a retrospective study conducted in 295 patients hospitalized for HF over a follow-up period of 461 days showed that hemoconcentration (defined as the highest quartile of hemoglobin change) was associated with reduced mortality only in univariable analysis, but not in multivariable analysis. This inconsistent data may result from differences in study populations, follow-up period, the definition of hemoconcentration used, a different model as well as small number of deaths [11]. In the current study we found hemoconcentration was associated with an improvement in survival compared with NC or hemodilution. Furthermore, in our study the stratification of patients into 3 groups according to the tertile of delta HCT as opposed to other classifications was deemed appropriate for the following reasons. First, hemodilution, NC, and hemoconcentration are part of a continuous spectrum of clinic states during the treatment of AHF. Previous research excluded NC or hemodilution, therefore the mortality rate in these subgroups were unknown. In fact, we found the mortality rate was significantly higher in patients with NC, or hemodilution than in those with hemoconcentration (31.8% vs. 18.3%, *P* = 0.013; 36.3% vs.18.3%, *P* = 0.001).

It is assumed that aggressive fluid removal may reduce renal perfusion and subsequently result in activation of the renin-angiotensin-aldosterone system (RAAS) and sympathetic nervous system (SNS), leading to cardiorenal syndrome. However, some studies have shown that hemoconcentration is associated with a significantly lower rate of all-cause death, despite its association with WRF [8, 9, 12]. The ESCAPE Trial was designed to evaluate the effects of aggressive decongestion on renal function and survival in 336 patients with decompensated HF [8]. The results of this trial revealed that aggressive fluid removal (hemoconcentration), even at the cost of WRF, may still positively affect survival. It is important to note that in this analysis, we did not find any difference in developing WRF during the course of hospitalization among the three groups. This result was consistent with a published study which reported that changes in volume status were not associated with the development of WRF [23]. Similarly, Ather et al. had also emphasized that changes in intravascular volume were not the leading cause of development of WRF [24].

A substantial portion of patients presented with stable, or increased volume status after treatment and were stratified into NC or hemodilution. Compared to hemoconcentration, hemodilution and NC carried a worse prognosis according to our data, however, there was no difference in risk of mortality between the two. Data regarding characteristics and outcomes on NC has been poorly studied so far. NC might represent a state of equilibrium between decongestion and congestion. The characteristics as well as prognosis of this population deserve further comprehensive analysis. Hemodilution is common during AHF treatment, and may result in pseudoanaemia [9], which could be attributed to many different precipitating factors, such as insufficient dosage of diuretics, diuretic resistance, disease deterioration etc.… Hemodilution may have a negative effect on patients with AHF by propagating poor peripheral oxygen delivery and the consequent ischemic damage to some organs [25]. In a prospective study by Androne et al. [25], hemodilution was assessed with I^131^-tagged albumin in 37 chronic HF patients. The study revealed that, over a 417-day period, the prevalence of hemodilution was as high as 46% and prognosis tended to be worse in patients with hemodilution than in those with true anemia. Similarly, Hong et al. reported that in AHF patients with severe renal dysfunction, new-onset anemia as a surrogate for hemodilution better predicted cardiovascular events than baseline anemia. Furthermore, hemodilution was associated with a higher incidence of two-year cardiovascular events [26]. Considering the poor outcomes associated with hemodilution and NC, our study supports that intensification of diuretic therapies is indicated in both groups.

### Limitations

This study was a retrospective observational single center study. Due to the observational nature of the study, it is impossible to confirm causality. Further, HCT change provided as a surrogate to direct measurements of plasma volume to assess for changes in volume status may lack specificity. Also, we were unable to rule out residual measured and unmeasured confounding factors that may result in different outcomes. Finally, longitudinal data on the change of relevant clinical or echocardiographic parameters (NT-proBNP, NYHA class, LVEF) over time was not available in our study. Also, data regarding diuretic dose was not reported in the study.

## Conclusions

Our findings indicate that a short-term change in HCT concentration is an independent risk factor for mortality in patients hospitalized with AHF. The top tertile of HCT change (ΔHCT > 1.5%) during hospitalization is associated with a lower risk of mortality. Patients with hemoconcentration experience improved outcomes compared those with NC and hemodilution, whereas mortality does not differ between those with NC and hemodilution. We therefore recommend the institution of more aggressive decongestive therapy in the acute phase. After discharge, patients with hemoconcentration may continue diuretic therapy or appropriately reduce their dosage. However, those with NC or hemodilution should undergo more aggressive diuresis. Additional, large-scale, prospective, randomized controlled studies are critically needed to confirm and characterize the association between short-term changes in HCT and prognosis and to develop optimal therapeutic strategies for volume management in AHF patients.

## Abbreviations

AHF: Acute heart failure
CI: Confidence interval
eGFR: Estimated glomerular filtration rate
HCT: Hematocrit
Hgb: Hemoglobin
HR: Hazard ratio
IQR: Interquartile range
LDL-c: Low density lipoprotein cholesterol
NC: No change
NT-proBNP: N-terminal pro-brain natriuretic peptide
NYHA: New York Heart Association
RAAS: Renin-angiotensin-aldosterone system
SNS: Sympathetic nervous system
WHO: World Health Organization
WRF: Worsening renal function
